# No Evidence That the Phoretic Mite *Poecilochirus carabi* Influences Mate Choice or Fitness in the Host Burying Beetle *Nicrophorus nepalensis*

**DOI:** 10.1002/ece3.71733

**Published:** 2025-07-04

**Authors:** Brendan Lan, Tanzil Gaffar Malik, Mu‐Tzu Tsai, Yi‐Ta Wu, Syuan‐Jyun Sun

**Affiliations:** ^1^ International Degree Program in Climate Change and Sustainable Development National Taiwan University Taipei Taiwan; ^2^ Department of Biology Duke University Durham North Carolina USA

**Keywords:** mate choice, phoresy, sexual selection, symbiosis

## Abstract

Mate choice is a fundamental aspect of sexual selection where the “chooser” chooses a “courter” by assessing a variety of traits that communicate potential fitness. However, the role of interspecific interactions, such as symbiosis, in shaping mate choice remains poorly understood. Here, we investigate whether phoretic mites *Poecilochirus carabi*, which can act as either mutualists or parasites, influence female mate choice or reproductive fitness in the burying beetle *Nicrophorus nepalensis* . These mites affect beetle fitness in context‐dependent ways, influenced by temperature, competitor presence, and mite density—factors that could potentially impact mate selection. In an olfactory‐based mate choice assay, we presented female *N. nepalensis* hosting a range of natural mite densities (0, 5, 10, or 20) with a choice between males carrying either 0 or 10 mites. Subsequently we allowed females to breed with their chosen male before evaluating the fitness effects of the varying male and female mite densities. We found no evidence that female *N. nepalensis* preferred males based on mite presence, regardless of their own mite density. Furthermore, mite density did not affect beetle fitness, as measured by brood size or average larval mass. However, mite reproductive output increased with higher total mite densities per breeding pair. Our findings suggest that, under naturally occurring conditions and in the absence of competitors, *P. carabi* mites do not influence female mate choice or beetle reproductive success in *N. nepalensis* .

## Graphical abstract

In this study, we tested whether phoretic mites (Poecilochirus carabi) influence female mate choice or reproductive fitness in the burying beetle *Nicrophorus nepalensis* . Despite the mites' context‐dependent effects on beetle success, we found no evidence that naturally occurring mite densities affect female preference, brood size, or larval mass. Our results suggest this symbiosis is ecologically neutral under benign conditions.

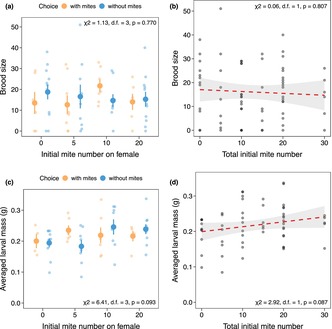

## 1 Introduction

Mate choice is a significant aspect of sexual selection that can influence the fitness of animals across sexually reproducing taxa (Andersson 1994; Rosenthal 2017; Ryan et al. 2007). Choosers assess potential mates' fitness through a variety of traits that are indicators of a potential mate's fitness. Choosing an “incorrect” mate can lead to a decrease in the potential fitness of the chooser. Most studies have focused on how mate choice is affected by traits that are either evaluated by the chooser or exhibited by the courter. For example, mate choice has been extensively studied in the context of ornamentation (Qvarnström et al. 2006), body size (López‐Cortegano et al. 2020), age (Beck and Powell 2000), mating history (Aich et al. 2020; Arnqvist and Nilsson 2000), ability to win bouts (Emberts et al. 2018; Emlen 2008), and motor performances (Byers et al. 2010) across taxa.

However, many traits that affect fitness have been overlooked as potential influencers of mate choice. For instance, symbiotic relationships such as mutualisms and parasitisms can increase or decrease an individual's fitness and have comparatively been seldom studied in the context of sexual selection. Among the studies that have examined how these symbiotic relationships may affect mate choice, parasitisms have been the obvious focus, especially in the context of parasite avoidance (Beltran‐Bech and Richard 2014; Ehman and Scott 2002; Reyes‐Ramírez et al. 2020) due to the more commonly intertwined relationship between host and parasite. For example, studies on fruit flies have shown male preference for unparasitized female mates (Markov et al. 2009; Wittman and Fedorka 2014); however, this appears to be context dependent (Arbuthnott et al. 2016). The effects of mutualisms on mate choice, although they may confer significant fitness benefits, have been comparatively understudied. This is likely because mutualistic associations are usually less intertwined than parasitisms (Bronstein 2001). However, burying beetles and their phoretic mites present a closely intertwined symbiotic system in which the mites act as protective mutualists or parasitic competitors that can have outsized fitness effects on their beetle hosts depending on the context (Beninger 1993; De Gasperin and Kilner 2015; Nehring et al. 2017; Sun and Kilner 2020; Wilson 1983; Wilson and Knollenberg 1987). Thus, this is an ideal system to test whether a complex symbiotic relationship has the potential to directly affect the mate choice in one of the symbiotic partners.

Burying beetles in the genus *Nicrophorus* are an intriguing subject of sexual selection studies as parents provide extensive care to their offspring—with many species showing high rates of offspring mortality in the absence of parental care, especially in early instar larvae (Trumbo 1992). In addition, individual *Nicrophorus* parents must work in tandem to compete against other carrion‐breeders such as blow flies (Diptera: Calliphoridae). Together, this means that a potential mate is extremely important for the survival of one's offspring and opens the door for sexual selection to be key in reproductive success. Indeed, mate choice studies in this genus examined the role that size (Beeler et al. 2002; Suzuki 2009), tendency to mate frequently (Hopwood et al. 2016), and rival presence (Suzuki 2009) play in mate choice. An overlooked aspect of their mate choice that remains unexplored is whether the density of their phoretic mites affects their mate choice.

Phoretic mites exert widespread and varied effects on their hosts that extend beyond mere phoresy, including context‐dependent parasitic or mutualistic interactions across different systems (Hodgkin et al. 2010; Seeman and Walter 2023). *Poecilochirus carabi* mites are common phoretic passengers on various *Nicrophorus* species, breeding in tandem with their host beetles on small carrion resources (Pukowski 1933). These mites have been experimentally shown to exhibit a mutualism–parasitism continuum in association with their burying beetle hosts (Sun and Kilner 2020). The burying beetles play the role of transportation, conferring a fitness benefit to *P. carabi* by acting as a flying shuttle to bring otherwise strictly cursorial mite passengers to the carrion on which both species breed. Once there, the mites will either increase beetle fitness by aiding beetles in competing with other carrion‐breeders, or compete with these beetles and their larvae directly for carrion, thus lowering beetle fitness (Sun et al. 2019). The mechanism by which mites increase burying beetle fitness has not been directly observed, but it is implied that mites likely directly prey upon competitor blow fly eggs (Springett 1968). However, in the absence of competitors like blow flies, mites lower burying beetle reproductive success, particularly at high mite densities (Nehring et al. 2017; Sun and Kilner 2020). In addition, the presence of mites can help smaller beetles overcome their competitive disadvantage when competing with larger conspecifics for access to carrion, increasing their fitness (Sun et al. 2019). Together, an individual beetle's phoretic mite density has direct fitness consequences for their offspring and thus has the potential to act as a trait that affects mate choice.

In this study, we consider how the presence of naturally‐occuring densities of *P. carabi* (hereafter “mites”) might influence mate preferences in female *Nicrophorus nepalensis* using olfactory‐based mate choice assays. Following mate‐choice assays, female burying beetles were allowed to breed with their chosen males to assess the fitness consequences of their choice. Larvae number and larvae weight were measured as indicators of beetle fitness. The number of mites was also measured to determine the degree of mite success. We hypothesize that female burying beetles prefer male beetles without mites rather than males with mites, and that females have a stronger preference to males without mites when the females carry higher initial mite densities themselves. In addition, in line with their mate choices, we predict that resulting pairs' total mite densities will have a negative relationship with burying beetle fitness.

## 2 Material and Methods

### 2.1 Study Species and Field Sampling

The experiments were conducted from April to May 2024. Both *N. nepalensis* burying beetles and *P. carabi* mites were cultured in the lab with ancestor individuals collected across six study sites (Datun Natural Park 25.1918° N, 121.5106° E, Fuzhou Shan Park 25.0163° N, 121.5549° E, Xianjiyan 24.9927° N, 121.5481° E, Luku Incident Memorial Park 25.0307° N, 121.6672° E, Huangdidian 24.9812° N, 121.6754° E, and Lishan 25.0228° N, 121.5957° E) in northern Taiwan in 2022. The maintenance of our beetle colony followed the protocol described by Malik et al. (2024). All beetles used for the olfactory‐based mate choice assay were bred in completely mite‐free conditions and were only used approximately 2 weeks following eclosion when they became sexually mature. The beetles used for the mate choice assay were fed small pieces of ground pork every 3–4 days prior to the trials.

To maintain the mite colony, we collected deutonymphs of *P. carabi* harvested from *N. nepalensis* individuals caught in the field. We bred the mite colony by mixing 3–5 individual mites from each different site until reaching a total of 20 mites per breeding container (14.2 diameter x 6.3 height cm), with a total of 10 containers. Each container was filled with moist soil to a depth of 1.5 cm, to which was added a pair of sexually mature beetles and a mouse carcass (20–30 g). The beetle pair and associated mites were then allowed to breed on the carcass. At dispersal, we collected all dispersing mites from the adult beetles and added them to a new container with another beetle, fed once a week with minced pork. We bred the mite colony once a month by introducing 20 mites from the most recent generation, again mixing 3–5 individual mites from different sources, with a total of 10 containers.

To assess the natural mite densities of *N. nepalensis* , we surveyed for the occurrence and natural densities of these beetles and their mites using hanging traps from October 2023 to May 2024, each baited with a commercial mouse carcass. In total, 1438 trapping events were conducted in northern Taiwan. Each fresh mouse carcass was left to decompose for 4 days prior to beetle and mite collection. Once captured, we separated the mites from the beetles using a fine brush and pairs of tweezers. We processed the beetles individually by sexing them and determining their body size by measuring the pronotum width to the nearest 0.01 mm with a vernier caliper. In addition, we counted all *P. carabi* mites associated with each beetle to determine mite density per beetle.

### 2.2 Experimental Setup

To set up the mate choice assay and the subsequent fitness assessment of breeding pairs, beetles were first measured upon sclerotization post‐eclosion. Measurements of the pronotum width were used as a proxy for beetle body size, as is the standard procedure (Hopwood et al. 2016). A total of 80 female beetles and 160 male beetles were used. Male beetles were matched into pairs that were from the same family and size‐matched so that there was less than a 0.6 mm (0.18 ± 0.15 mm; mean ± SD) pronotum width difference between individuals. These matchings were done to minimize variations caused by genetics and by size. Following this, each of the resulting male pairs were matched with individual females, ensuring that the male pairs were not genetically related to the females. These trios were then randomly assigned to four treatment groups (*n* = 20 trios per treatment; Table S1). The densities of phoretic mites traveling on these beetles in situ vary, with previous sampling discovering that 84.4% of wild *N. vespilloides* carried between 0 and 20 *P. carabi* mites (Sun et al. 2019) and a similar distribution was found for *N. nepalensis* (Figure S1). Following this, we experimentally applied various naturally occurring mite densities to female beetles in the following four treatments: 0 mites, 5 mites (low density), 10 mites (medium density), 20 mites (high density) (Table S1). Each male within a matched male pair was either treated with 0 mites or 10 mites. Mites were applied to these beetles from the captive mite colony using forceps and brushes. When mites were transferred onto each beetle, the mites were allowed to settle onto the beetle for approximately 2 h prior to mate choice assays.

To simulate breeding conditions and encourage natural mate choice, a 22–30 g mouse carcass was introduced alongside each male in the holding chamber of the mate choice arena (Figure S2) similar to the preference trials used in Delclos et al. (2021). These frozen–thawed mouse carcasses were sourced from a commercial feeder mouse seller (Mice Plus, Changhua, Taiwan) intended for consumption by pet reptiles. Each carcass was weighed prior to use and size‐matched within 1 g (0.36 ± 0.26 g; mean ± SD) to create size‐matched pairs of carcasses. These carcass pairs were then randomly assigned to beetle trios and left outside for 30 h to begin decomposition prior to mate choice assays, making them more attractive to breed upon (Kalinová et al. 2009).

### 2.3 Mate Choice Assay

An olfactory‐based mate choice assay was conducted, because burying beetle mate choice and reproduction is likely olfactorily mediated as male burying beetles release pheromones (“calling”) to attract females (Beeler et al. 1999; Trumbo and Eggert 1994). Additionally, other key behaviors such as locating a carcass (Kalinová et al. 2009) and nestmate recognition (Steiger et al. 2008) are also olfaction‐based. The mate choice of females was studied rather than males, because female *Nicrophorus* species contribute more towards the parental care of offspring (Hwang and Lin 2013; Scott and Traniello 1990) indicating that they are likely to be the more “choosier” sex. This is supported by the fact that females seem pickier in other *Nicrophorus* species as well, sometimes refusing to copulate with males (Suzuki 2009).

For each trial the female was first placed in the acclimation chamber while the two males and their respective mouse carcasses were placed in their own holding chambers (Figure S2a). Throughout the entire trial, including acclimation, air movement was powered by an air sampling pump (SKC XR 224‐PCXR8, SKC Inc., USA) and volatiles were allowed to pass through the arena as indicated by Figure S2a. The airflow was measured to be 19 mL/min. Each trial was done in the dark under red lights (Figure S2b,c) to simulate a nighttime environment and conducted at approximately 21°C. The female was held in the acclimation chamber for at least 5 min before commencing the trial. While acclimation was occurring, the female was exposed to pheromones from both males. After acclimation, the female was allowed to freely roam the arena by sliding up the removable door. During this time, if the female entered the chamber adjacent to the holding chamber where only a single male could be smelled and decided to make physical contact with the choice wall (Figure S2a), the male beetle in the associated holding chamber was determined to be her mate choice. Females did not often leave the holding chamber after making physical contact with the choice wall. If the female did not contact a choice wall within 20 min post‐acclimation, she was determined to have not made a choice and was not allowed to breed. This methodology in determining female choice is similar to previous work on burying beetle mate choice (Beeler et al. 2002). Mate choice assays were conducted at 5:30 pm each day, as that is a time at which beetles are active. The arena was made from laser‐cut acrylic. Up to four trials were conducted at the same time (Figure S2c).

### 2.4 Fitness Assessment

Of the 80 females tested, 69 made a choice within the 20‐min assay period. Immediately upon the female beetle making a choice in the assay, the female, her chosen male, and all mites on the beetles were removed from the mate choice assay and allowed to breed on the carcass in a cylindrical plastic container (14.2 cm diameter × 6.3 cm height). This breeding container had a soil depth of approximately two centimeters and was kept at a mean ambient air temperature of 17.8°C. This temperature reflects their natural breeding season in the wild and is the standard used in laboratory rearing protocols (Malik et al. 2024). These containers were darkened with an opaque covering to encourage natural breeding events of burying beetles.

Approximately 90 h after the burying beetles were allowed to breed, the number of eggs were counted as a measure of fitness. However, it was imperative to count the egg number without disturbing the parents. As such, only the number of eggs that were visible from the bottom of the cylindrical container were counted as a proxy for the total number of eggs each female laid (Malik et al. 2024). Burying beetles typically lay their eggs at the bottom of their enclosures, and thus most eggs are visible from the bottom. However, as this is only a proxy measurement of the true egg count, larval measurements were taken as well.

Upon larval dispersal, the brood size, the weight of each brood, and the number of mites were measured. Out of the 69 breeding cohorts, five experienced complete breeding failure with no eggs laid, and six produced no offspring. This resulted in a total of 58 broods with available larval fitness data. Larval dispersal typically occurred approximately 12 days (12.3 ± 0.8 days; mean ± SD) after the onset of breeding. The number of larvae was counted manually, and total larval mass was measured with a precision scale to the nearest 0.001 g (Shimadzu; Model: ATX224R). Thus, we calculated the averaged larval mass by dividing total larval mass by the number of larvae.

A benchtop counter was used to determine the number of phoretic mites on both parent beetles post‐reproduction (upon larval dispersal). This number was used as a proxy for the total number of mite offspring (the number of mites post‐breeding). Within 24–48 h of *Nicrophorus* arriving at a carcass in the wild, the mites on their bodies disperse from the beetles' bodies and molt into their adult stage (Brown and Wilson 1994). In their adult stage, these mites are not phoretic (Brown and Wilson 1994), thus all mites on the adult bodies post‐reproduction are the offspring of the original mites rather than the original mites themselves. Additionally, most mite offspring (~90%) tend to disperse with the adult beetles rather than waiting for the next generation of beetles to pupate and develop (Schwarz and Müller 1992). As such, counting the number of mites on both beetles post‐reproduction was deemed an acceptable measure of the number of mite offspring.

### 2.5 Statistical Analysis

To analyze female burying beetle mate choice between males with and without mites, we performed binomial tests using the “binom.test” function for each mite density treatment (0, 5, 10, and 20 mites) of female beetle to determine if the proportion of females choosing males with mites was significantly different from 0.5, which would indicate a lack of preference.

To analyze the effects of mite density treatment and mate choice on the fitness consequences of beetles, we used a generalized linear mixed model (GLMM) to analyze the brood size with a negative binomial distribution to account for data overdispersion. Female mite density treatment (0, 5, 10, and 20 mites) and the female mate choice (males with or without mites) were included as categorical variables, whereas female body size and carcass mass were included as covariates. In addition, we analyzed the brood size in another model, in which we considered the total number of mites (mites on both the male and the female) as a continuous variable, wherein female body size and carcass mass were also included as covariates. To analyze the average larva weight per brood (hereafter “averaged larval mass”), we used a GLMM with a gaussian distribution. Similar to analyzing brood size, we included female mite density treatment and the female mate choice as categorical variables, female body size and carcass mass as covariates. Additionally, we included brood size as a covariate since brood size has been known to negatively affect averaged larval mass in burying beetles (Schrader et al. 2015). We also included days to dispersal as a covariate to account for potential effects of developmental timing. In addition, we analyzed the averaged larval mass in another model, in which we considered the total number of mites (mites on both male and female beetles) as a continuous variable, wherein female body size, carcass mass, and the brood size were included as covariates.

To analyze the effects of mite density on the fitness consequences of mites, we used a GLMM to analyze the number of mite offspring with a negative binomial distribution. The total number of mites was included as a continuous variable, whereas female body size and carcass mass were included as covariates. To determine the relationship of fitness consequences between beetles and mites, we used GLMMs to analyze the brood size and the averaged larval mass with a negative binomial distribution and Gaussian distribution, respectively. In both models, we included the number of mite offspring, female body size, and carcass mass as covariates, and additionally included the brood size when analyzing the averaged larval mass.

All statistics were conducted in R statistical software version 4.1.2 (R Core Team 2024). We evaluated all model residuals for normal distribution and overdispersion using the *DHARMa* package version 0.4.7 (Hartig et al. 2024). GLMMs were analyzed using the “glmer” function in the *lme4* package version 1.1.35.5 (Bates et al. 2015). We obtained *p* values for the main effects using the “Anova” function in the *car* package version 3.1.3 (Fox et al. 2001). In all GLMMs, the family origin of female beetles was included as a random factor to account for this variation since multiple females from the same families were used in the experiment.

## 3 Results

Of 1438 trapping events, 528 traps caught at least one beetle. In these traps, the number of beetles per trap ranged from 1 to 18, with an average of 2.8 beetles per trap. Our field trapping further showed that 72.0% (1080 out of 1499 individuals) of *N. nepalensis* individuals carried at least one individual *P. carabi*. Notably, males were more likely than females to host mites (χ^2^ = 5.41, d.f. = 1, *p* = 0.020), with 74.6% of males and 69.3% of females carrying at least one mite. The number of mites per individual ranged from 0 to 166, with an average of 8 mites. The mean and frequency distribution of mite numbers were similar between males and females (mean: t = −0.61, *p* = 0.540; distribution: *D* = 0.05, *p* = 0.240; Figure S1).

**FIGURE 1:**
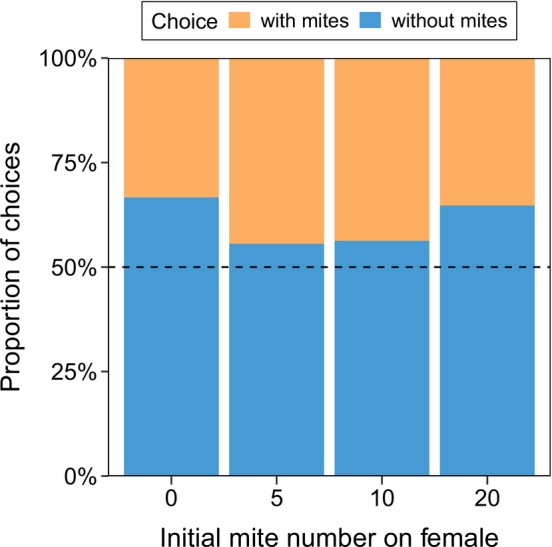
The number of mites on the female had no effect on her choice of male mate in all mite density treatments. The dashed line indicates no preference.

**FIGURE 2:**
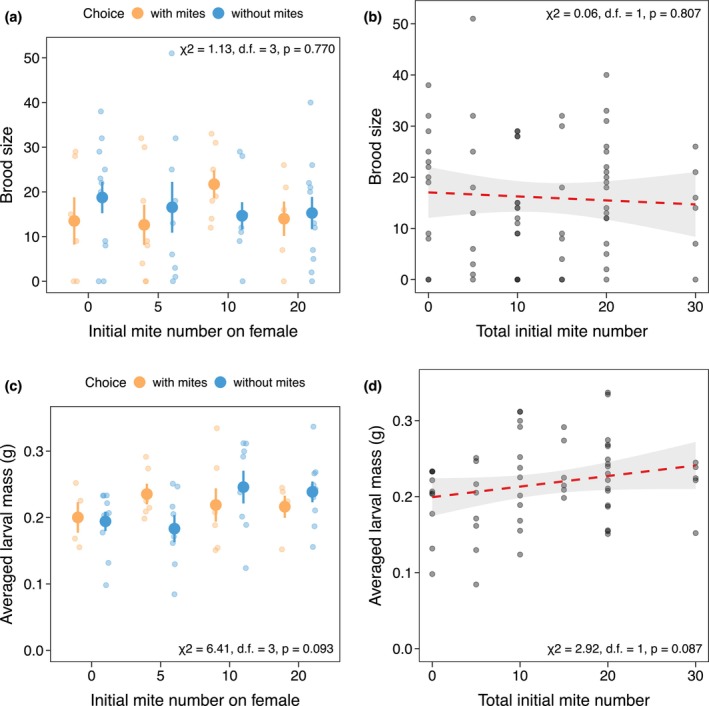
The effects of the initial mite number on just the female (a, c) and the total mite number of each breeding cohort (b, d) on the brood size (a, b) and averaged larval mass of each brood (c, d). (a) The effect of the initial mite number of just the female beetle on brood size. (b) The effect of total mite number of each breeding cohort on brood size. (c) The effect of initial mite number of just the female beetle on the averaged larval mass of each brood. (d) The effect of total mite number of each breeding cohort on the averaged larval mass of each brood.

There was no significant preference in female beetles for male beetles with or without mites across all female mite densities tested (0 mite: *p* = 0.238; 5 mites: *p* = 0.815; 10 mites: *p* = 0.804; 20 mites: *p* = 0.332; Figure 1). In line with this lack of significant preference in mate choice, different initial mite numbers of just females and total mite numbers in each cohort both had no effect on the brood size (χ^2^ = 1.13, d.f. = 3, *p* = 0.770; Figure 2a; χ^2^ = 0.06, d.f. = 1, *p* = 0.807; Figure 2b) and the averaged larval mass (χ^2^ = 6.41, d.f. = 3, *p* = 0.093; Figure 2c; χ^2^ = 2.92, d.f. = 1, *p* = 0.087; Figure 2d).

**FIGURE 3:**
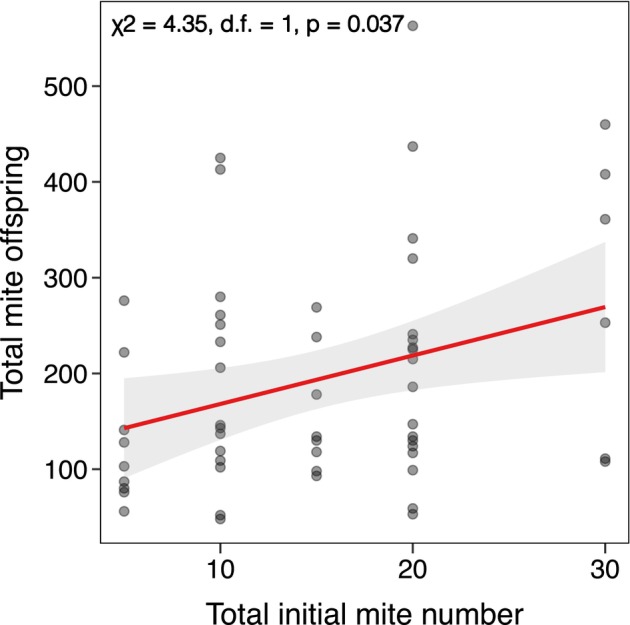
The total initial number of mites per breeding cohort had a positive effect on the total number of mite offspring. The solid line indicates statistically significant relationship from GLMMs, whereas the shaded area represents 95% confidence interval.

**FIGURE 4:**
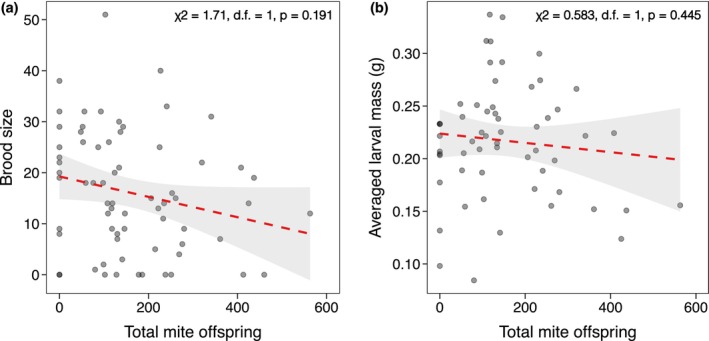
The total number of mite offspring resulting from each breeding cohort had no effect on brood size (a) or the averaged larval mass in each brood (b). The dashed lines indicate statistically non‐significant relationships from GLMMs, whereas the shaded areas represent 95% confidence intervals.

Although the starting number of total mites in each breeding cohort did have a positive relationship with the number of mite offspring (χ^2^ = 4.35, d.f. = 1, *p* = 0.037; Figure 3), the mite offspring number did not explain beetle larvae fitness as the number of individuals in a brood (χ^2^ = 1.71, d.f. = 1, *p* = 0.191; Figure 4a) or averaged larval mass (χ^2^ = 0.583, d.f. = 1, *p* = 0.445; Figure 4b).

## 4 Discussion

Our findings suggest that mites, at most naturally occurring densities, do not affect burying beetle mate choice or fitness. Given that we did not find any effect of mite density on burying beetle fitness, the lack of a mate preference for males without mites is in line with the lack of a mite‐induced fitness effect. Although the starting mite densities in each pairing did positively affect the number of total mite offspring per cohort (Figure 3), burying beetle fitness remained unaffected (Figure 4). However, this relationship is likely modulated by other biotic and/or abiotic factors, which could, in turn, influence mate choice in burying beetles. Based on these results, we conclude that mites are largely benign symbionts and found no evidence that they influence mate choice in *N. nepalensis* .

In agreement with these findings, we also found that wild males were more likely to carry mites than wild females—thus it would be costly for females to search specifically for males without mites. A prolonged mate search driven by a preference for mite‐free males could delay copulation and preparation of carrion, a rare and ephemeral resource. Therefore, other more salient factors may be influencing female mate choice, or mites may only impact *N. nepalensis* mate choice in contexts where reproductive success is more drastically affected.

Mate choice in *Nicrophorus* has been investigated broadly in the past as parent beetles of many species in this genus provide extensive biparental care. Thus, the choice of a potential mate is extremely important. As mentioned above (see “Introduction”), mate choice studies in this genus have often focused on the role that size plays in burying beetle mate choice (Beeler et al. 2002; Suzuki 2009). Beetle size is a reliable proxy for fitness because larger beetles win fights for access to carrion more often (Otronen 1988) and because larger beetles are more fecund (Scott 1998). The presence of mites can also affect fitness both positively and negatively. For example, mites contextually increase beetle fitness by aiding smaller beetles in intraspecific fights (Sun et al. 2019) or by outcompeting blow flies on carrion (Sun and Kilner 2020). Conversely, mites can also decrease beetle fitness by competing directly with beetle larvae in the absence of competitors (Nehring et al. 2017; Sun and Kilner 2020). Thus, beetles might be able to detect mite loads on potential mates and make mating decisions accordingly. This investigation is the first which examines whether these phoretic mites—capable of modulating beetle fitness—affect mate choice.

Our study found no evidence that females altered their mate choice based on mite presence when males were size‐matched (Figure 1). However, we could not test whether male size might mediate this relationship. Prior studies suggest that female *Nicrophorus* prefer larger males (Beeler et al. 2002), but smaller males carrying mites may mitigate size‐related disadvantages through enhanced success in contests (Sun et al. 2019). It is therefore possible that mite presence could influence female preference for male size, though this hypothesis remains untested in our study. In addition, it is unknown whether burying beetles can olfactorily or otherwise detect the mite densities of intraspecifics and make mate choices based on that information. However, given the significant fitness consequences that mites can exert (Nehring et al. 2017; Sun and Kilner 2020), there is likely selective pressure for such detection mechanisms. We assume the mechanism for mite detection to be, at least in part, olfactorily mediated due to the centrality of olfaction in *Nicrophorus* spp. (Beeler et al. 1999; Kalinová et al. 2009; Steiger et al. 2008; Trumbo and Eggert 1994), but this remains speculative and beyond the scope of our current investigation.

Previous work found that mites acted as parasites in the absence of other competitors (Nehring et al. 2017); however, in this current investigation, mites failed to impact burying beetle fitness. This discrepancy is likely due to one or both of the following factors: the temperature our cohorts were reared in and the species used for this experiment. When considering temperature, this investigation allowed cohorts of burying beetles and mites to breed in incubators that were kept at a mean of 17.8°C. In Sun and Kilner (2020), three temperatures were used to rear beetles with varying numbers of mites: 11°C, 15°C, and 19°C. The beetles in the 19°C rearing treatment seemed to show the least negative fitness effect as mite density increased. Nehring et al. (2017) reported a negative relationship between mite number and burying beetle fitness at 20°C. Additionally, both studies used mite densities well within the range of the current investigation: 0, 10, and 20 mites per beetle pair in Sun and Kilner (2020) and 10 mites per pair in Nehring et al. (2017). Our current investigation had cohorts with 0, 5, 10, 15, 20, and 30 mites (Table S1). Thus, it is unlikely that mite densities, at least within most naturally occurring contexts, modulate this relationship. Taken together, it is probable that this association between burying beetles and mites, with respect to reproductive success, is quite sensitive to temperature, ranging from a commensal association to a parasitic one, with mites negatively affecting burying beetle fitness at the two ends of the temperature gradient.

To our best knowledge, our study is the first empirical examination of mate choice and mite association in *N. nepalensis* , and it is possible that species‐specific dynamics influence the outcome. Previous research has shown that *N. vespilloides* , *N. vespillo* , and *N. tomentosus* respond differently to mite interactions (Nehring et al. 2017; Wilson and Knollenberg 1987). Future research should investigate how temperature and species identity interact to shape the fitness effects of mites across the *Nicrophorus* genus. In particular, studies involving beetles of varying body sizes could help clarify whether mites modulate mate choice under parasitic conditions. Prior research on the role of parasites in mate choice across taxa has revealed highly variable outcomes (Cantarero et al. 2022; Ehman and Scott 2002; Reyes‐Ramírez et al. 2020), suggesting a complex interplay that may also apply to this system.

Although mites seem to be protective mutualists of burying beetles, this relationship only occurs in the presence of competitors such as blow flies (Springett 1968; Sun and Kilner 2020; Wilson 1983). It is possible that, in the presence of blow flies, burying beetles will preferentially choose mates with more mites to increase their fitness, because Sun and Kilner (2020) showed that as mite numbers increased, burying beetle fitness also increased in the presence of blow fly competitors. This relationship was reported to be affected by temperature (Sun and Kilner 2020), and thus temperature seems to be key to this symbiosis, both in the presence and absence of competitors.

From the perspective of the mites, our results indicate that initial mite densities of each cohort strongly influenced their reproductive success (Figure 3), especially given that an average of 8 mites were found on wild beetles (Figure S1) and that we used initial mite densities up to 30 in each cohort (Table S1). This indicates that the range of initial mite densities tested do not reach the carrying capacity of the carcass in our conditions, even with burying beetles breeding upon them in tandem. Given that more than two beetles were often attracted to and bred upon one carcass as indicated by our field results, wild mites likely experience a wider range of initial mite numbers than tested in this investigation. Further increasing the initial number of mites would likely negatively affect mite reproductive success and potentially negatively affect burying beetle fitness.

Overall, this investigation found no evidence that naturally occurring densities of *P. carabi* influence *N. nepalensis* mate choice (Figure 1) or reproductive success in the absence of other competitors (Figure 2). Even as mite number increased, mite reproductive success (Figure 3), burying beetle fitness remained unaffected (Figure 4). The potential fitness effects of mites on *N. nepalensis* are likely modulated by temperature and the presence of competitors, as is the case with other *Nicrophorus* species (Nehring et al. 2017; Sun and Kilner 2020). However, whether mate choice in the presence of mites is affected by a shift in their contexts, such as different temperatures and/or the presence of competitors that cause mites to act as parasites or as mutualists remains an open and intriguing question.

## Author Contributions

**Brendan Lan:** conceptualization (equal), data curation (lead), formal analysis (equal), investigation (lead), methodology (lead), software (equal), validation (lead), visualization (equal), writing – original draft (lead), writing – review and editing (equal). **Tanzil Gaffar Malik:** conceptualization (equal), data curation (equal), investigation (equal), methodology (equal), resources (equal), validation (equal), visualization (equal), writing – original draft (equal), writing – review and editing (equal). **Mu‐Tzu Tsai:** data curation (equal), investigation (equal), methodology (equal), resources (equal), validation (equal), visualization (equal), writing – original draft (equal), writing – review and editing (equal), writing – review and editing (equal). **Yi‐Ta Wu:** data curation (equal), investigation (equal), methodology (equal), resources (equal), validation (equal), writing – original draft (equal), writing – review and editing (equal). **Syuan‐Jyun Sun:** conceptualization (equal), data curation (equal), formal analysis (lead), funding acquisition (lead), investigation (lead), methodology (lead), project administration (equal), resources (lead), software (lead), supervision (lead), validation (equal), visualization (equal), writing – original draft (equal), writing – review and editing (equal).

## Conflicts of Interest

The authors declare no conflicts of interest.

## Supporting information


Data S1.

## Data Availability

Data and code for this research work have been archived within the Zenodo repository https://zenodo.org/records/15171551.
