# Supplementary material for: No Evidence That the Phoretic Mite Poecilochirus carabi Influences Mate Choice or Fitness in the Host Burying Beetle Nicrophorus nepalensis
Source: Ecol Evol. 2025 Jul 4;15(7):e71733. doi: 10.1002/ece3.71733 (PMC12231222; doi:10.1002/ece3.71733)
Supplement: Supplementary file 1 — Data S1. [file ECE3-15-e71733-s001.docx]

**Supporting Information**

**Table S1.** A summary of the treatment groups used in the mate choice studies and subsequent fitness assessments.

| Treatment | Female Beetles | Male Beetles | Resulting Mite Numbers per Cohort |
| --- | --- | --- | --- |
| 1 (n=20) | 0 mites | 0 mites | 0 mites |
|  |  | 10 mites | 10 mites |
| 2 (n=20) | 5 mites | 0 mites | 5 mites |
|  |  | 10 mites | 15 mites |
| 3 (n=20) | 10 mites | 0 mites | 10 mites |
|  |  | 10 mites | 20 mites |
| 4 (n=20) | 20 mites | 0 mites | 20 mites |
|  |  | 10 mites | 30 mites |
| Total: n=80 |  |  |  |

**
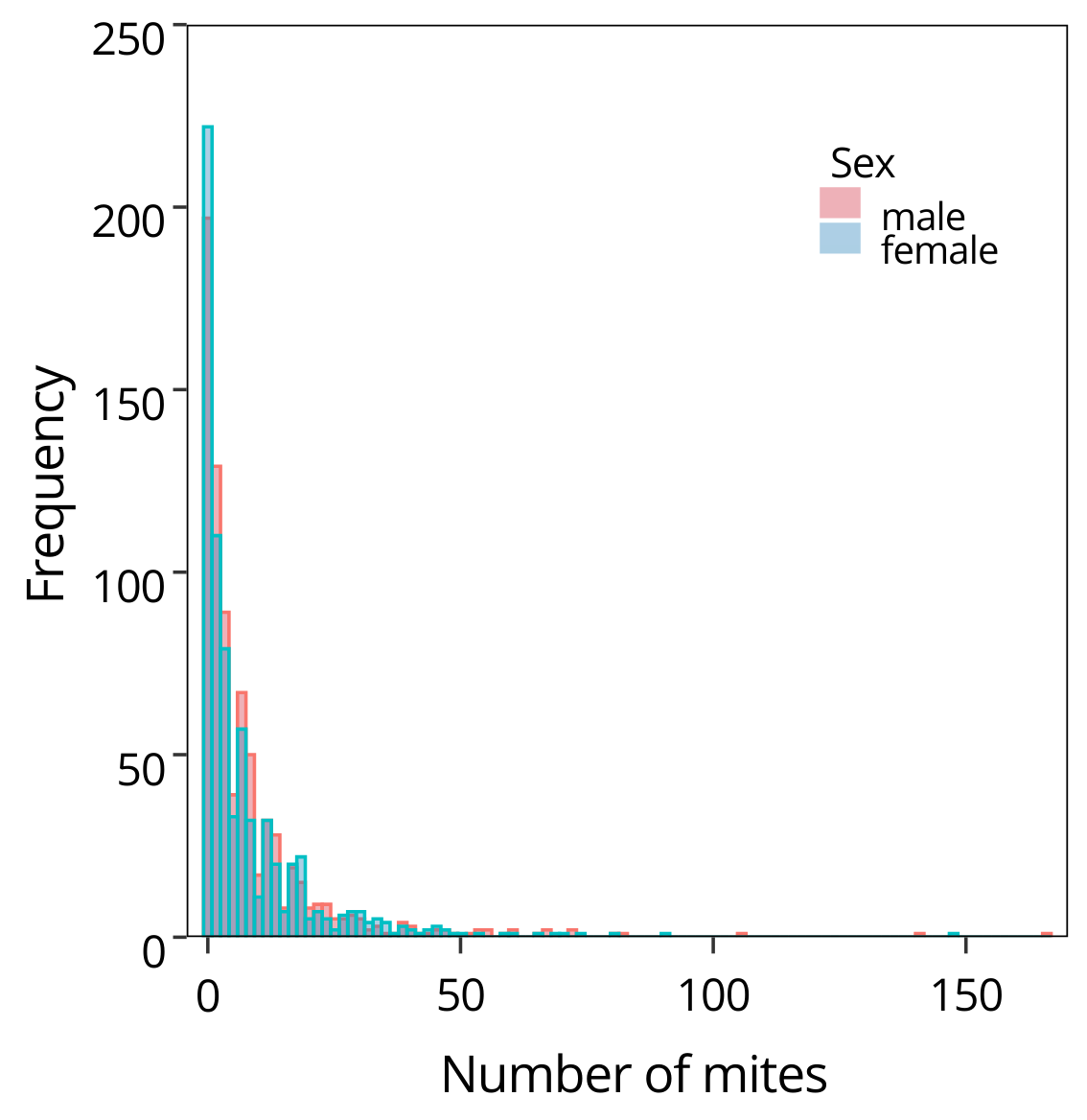
**

**Fig S1.** Histogram depicting results from counting the mites on field-trapped burying beetles. The mite number per individual ranged from 0 to 166, with an average of 8 mites.


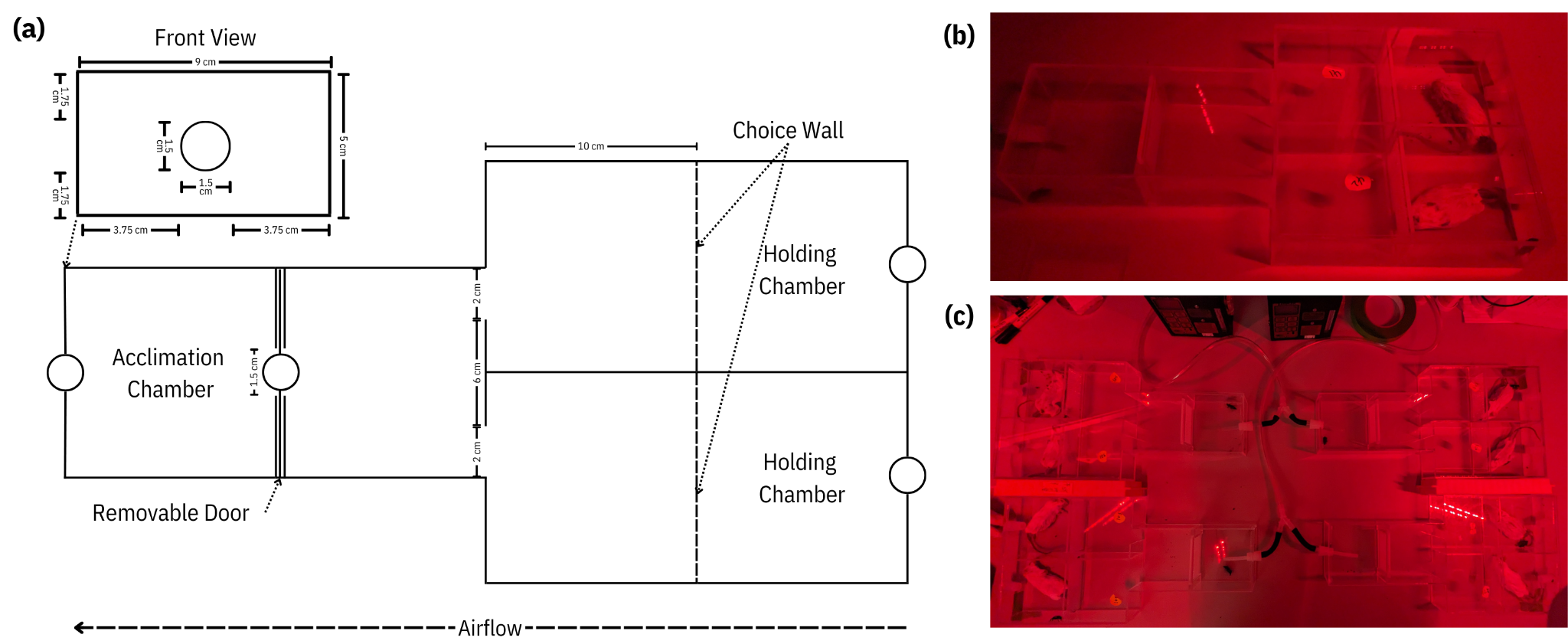


**Fig S2.** (a) Diagram of the mate choice arena depicting the Acclimation Chamber, Removable Door, Choice Walls, and Holding Chambers. The choice wall is perforated as indicated by the dashed line. Airflow moves as indicated by the dashed line arrow. The removable lid is not depicted here. (b) A photo of a mate choice arena in use with the female in the acclimation chamber, and both males with their respective carcasses in their holding chambers. (c) A photo of four mate choice arenas being utilized at once. In (b,c), the experiments were conducted under red light conditions.
